# Symmetry of the Neck Muscles’ Activity in the Electromyography Signal during Basic Motion Patterns

**DOI:** 10.3390/s23084170

**Published:** 2023-04-21

**Authors:** Gabriela Figas, Anna Hadamus, Michalina Błażkiewicz, Jolanta Kujawa

**Affiliations:** 1 Clinic of Medical Rehabilitation, Medical University of Lodz, 90-419 Lodz, Poland; gabriela.figas@umed.lodz.pl (G.F.); jolanta.kujawa@umed.lodz.pl (J.K.); 2 Department of Rehabilitation, Faculty of Dental Medicine, Medical University of Warsaw, 02-091 Warsaw, Poland; 3 Faculty of Rehabilitation, The Józef Piłsudski University of Physical Education in Warsaw, 00-968 Warsaw, Poland; michalinablazkiewicz@gmail.com

**Keywords:** cervical spine, surface electromyography, symmetry, sternocleidomastoid, upper trapezius

## Abstract

The activity of muscles during motion in one direction should be symmetrical when compared to the activity of the contralateral muscles during motion in the opposite direction, while symmetrical movements should result in symmetrical muscle activation. The literature lacks data on the symmetry of neck muscle activation. Therefore, this study aimed to analyse the activity of the upper trapezius (UT) and sternocleidomastoid (SCM) muscles at rest and during basic motions of the neck and to determine the symmetry of the muscle activation. Surface electromyography (sEMG) was collected from UT and SCM bilaterally during rest, maximum voluntary contraction (MVC) and six functional movements from 18 participants. The muscle activity was related to the MVC, and the Symmetry Index was calculated. The muscle activity at rest was 23.74% and 27.88% higher on the left side than on the right side for the UT and SCM, respectively. The highest asymmetries during motion were for the SCM for the right arc movement (116%) and for the UT in the lower arc movement (55%). The lowest asymmetry was recorded for extension–flexion movement for both muscles. It was concluded that this movement can be useful for assessing the symmetry of neck muscles’ activation. Further studies are required to verify the above-presented results, determine muscle activation patterns and compare healthy people to patients with neck pain.

## 1. Introduction

The common and most widely acknowledged method of accessing muscle activation and therefore analysing its function is surface electromyography (sEMG). It evaluates nerve–muscle activity during static conditions and active movements [1]. There are no reference data of valid resting muscle tone. It strongly depends on the cross-section of the examined muscle, the thickness of the subcutaneous tissue and skin resistance, which varies between individuals [2]. The signal collected during motion can be compared either to the maximum signal level collected during maximum voluntary contraction (MVC) [3] or to the maximum value from the same recording as analysed [4,5]. This gives the opportunity to compare muscles and persons with each other. One of the advantages of surface electromyography is its non-invasive character, which allows physiotherapists to use it without additional qualifications, which are needed for needle EMG. However, the usage of sEMG is limited to the superficial muscles [6]. In the neck region, sEMG can be used to assess trapezius [6] and sternocleidomastoid muscles [2], although some researchers also measured the anterior scalene and neck extensors [7,8].

The sternocleidomastoid muscle (SCM) is one of the largest and most superficial cervical muscles. The primary actions of the muscle are rotation of the head to the opposite side, lateral flexion to the same side and flexion of the neck. The descending part of the trapezius muscle—the upper trapezius muscle (UT)—is responsible for the extension of the neck and bending of the head and neck to the side [9,10]. Proper coordination and cooperation between those two muscles are necessary for correct head and neck movements. As superficial muscles, they are both located at a greater distance from the joint centre and therefore work on a larger moment arm than deep muscles. Therefore, they are both able to control the cervical spine “en bloc” to counteract external forces, as well as to control head load during movements [11,12]. These muscles were selected for this study as they are very important for controlling the movements of the cervical spine during functional activities [13]. Additionally, the upper trapezius and sternocleidomastoid muscles are often involved in work-related musculoskeletal disorders of the upper arms [14].

The analysis of multiple cases shows the increase in bioelectrical signals in both rest and movement in patients with back or neck pain [1]. In patients with cervical pain, UT shows higher activity during a functional task of the upper extremity and a lower ability to relax after finishing the task. The higher activity also applies to SCM and scalene muscles [13]. Due to the fact that the muscles of the cervical area provide nearly 80% of the mechanical stability of this region and, on the other hand, are responsible for high mobility in three planes of motion, even the smallest imbalance in muscle activity can have a great impact on the head and neck movements. Such a disorder may, in turn, affect the onset of complaints about the neck region [15]. In cervical pain patients, postural asymmetry can be observed [16]. Such asymmetry affects motor strategies and muscle activation patterns and increases the risk of injury [17]. Additionally, patients with neck pain demonstrate disturbed motor control during the performance of a functional activity [13,16]. Nowadays, one of the most common deviations of correct posture is forward head posture (FHP), which is characterized by hyperextension of the upper cervical spine and flection of the lower cervical spine. Changes occur in the sagittal plane and can be observed from a side view as a head protraction, which shifts the centre of gravity forward [18]. Hyperextension of the upper cervical spine is a typical compensatory mechanism to maintain the horizontality of the sight [19]. FHP results in the shortening of cervical extensors, including UT, which leads to an increased moment arm to counteract the weight of the head [20]. Sternocleidomastoid muscles are also affected [21]. Both UT and SCM, as well as other neck muscles, show decreased activity during neck motions [22]. The muscle imbalance induced by FHP can result in disturbed activation of the additional muscles needed to maintain neck and head posture and decreased muscular efficiency [23]. Although forward head posture is a rather symmetrical disorder, it strongly affects muscles’ function, both at rest and during movements.

There are only a few reports analysing the work of paraspinal muscles during motion, most of them concerning the lumbar spine [1]. Simultaneously, the literature lacks data on the symmetry of neck muscle activation [24]. Under physiological conditions, the activity of the muscles during symmetrical movements such as flexion or extension should be symmetrical. Additionally, during rotation movements, symmetrical work is dominant, while during lateral movements, the muscles work asymmetrically [25]. Nevertheless, muscle activation during motion in one direction should be symmetrical when compared to the activity of the contralateral muscles during motion in the opposite direction [1].

Due to the issues described above, it is important to define what is the proper activation of the neck muscles, both at rest and during movements. This will allow us to define the reference values to be used in the clinical assessment of patients with neck pain. Additionally, muscles’ activation symmetry should be defined to set its reference values.

Therefore, this study aimed to analyse the activity of the upper trapezius and sternocleidomastoid muscles at rest and during basic motions of the neck, as well as to determine the symmetry of the muscle activation. Three hypotheses were stated before the study: (1) the rest activity of the neck muscles should be lower than 10% of the maximum voluntary contraction (MVC) value and it should be symmetrical, (2) symmetrical movements should produce symmetrical activation of the measured muscles and (3) asymmetrical movements should affect asymmetrical activation of the muscles.

## 2. Materials and Methods

### 2.1. Participants

The study group included 18 right-handed participants (9 men and 9 women) aged from 24 to 68 years old without significant neck disability (Table 1). Sample size calculation was performed based on EMG data collected previously by one of the authors (A.H.) and partly published by Wiaderna et al. [26]. The required sample size was 14 for ANOVA test power > 0.8. The inclusion criteria comprised age between 18 and 70 years old, and a Neck Disability Index result lower than 15 points. The exclusion criteria included hypermobility syndrome (assessed in the physical examination and with the Beighton Score); any history of major rheumatic, orthopaedic (including spinal scoliosis), or neurological disease, as well as any other known condition that could adversely affect cervical neuromuscular function; and any current complaints in the neck or other spine levels. All the participants agreed and signed the written consent to take part in this study.

The project was approved by the Bioethics Committee of the Medical University of Lodz (RNN/115/21/KE, approved 11 May 2021) and conducted in accordance with the Declaration of Helsinki for research involving human subjects.

### 2.2. Protocol and EMG Data Acquisition

This study is part of a bigger project called VRneck SOLUTION, carried out from December 2020 to November 2023. The results presented below are planned to be used as reference values for evaluating the VR training system in patients with neck disorders. Therefore, a cross-sectional design was applied.

All the measurements were performed between 9 AM and 5 PM. Participants were advised to avoid caffeine, physical exercises and heavy load lifting for 24 h before the measurements. All the participants were qualified according to the inclusion and exclusion criteria by a medical doctor with a specialization in rehabilitation. EMG measurements were performed by a qualified physiotherapist.

Two muscles were measured bilaterally using surface electromyography: the sternocleidomastoid muscle and the descending part of the trapezius muscle. The EMG signal was acquired at a sampling frequency of 1000 Hz with the 4-channel eMotion EMG system (Bittum Biosignals Oy, Kuopio, Finland). Ag/AgCl ECG electrodes (Covidien Kendall H92SG, KD Medical GmbH, Berlin, Germany) were cut and placed on the skin according to SENIAM standards [6] at a 20 mm distance. Reference electrodes were placed on the spinal process of C_7_ and Th_1_ for the left and right trapezius, respectively, and in the middle of the clavicle for the sternocleidomastoid muscle.

**Figure 1:**
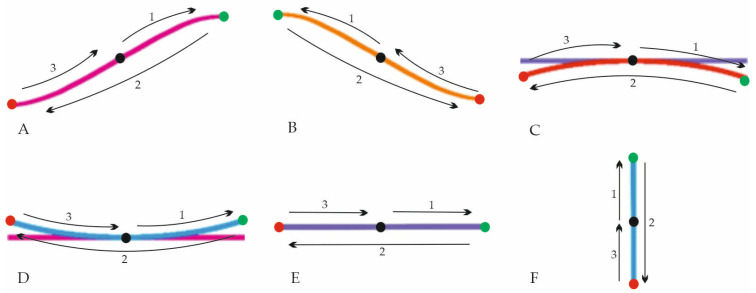
Motion patterns in the VRneck system: (**A**)—right arc, (**B**)—left arc, (**C**)—bottom arc, (**D**)—upper arc, (**E**)—rotation and (**F**)—extension–flexion movement; the direction and order of movements are indicated by arrows. Black dot—starting and ending point, green dot—goal of the first movement, red dot—goal of the second movement.

**Figure 2:**
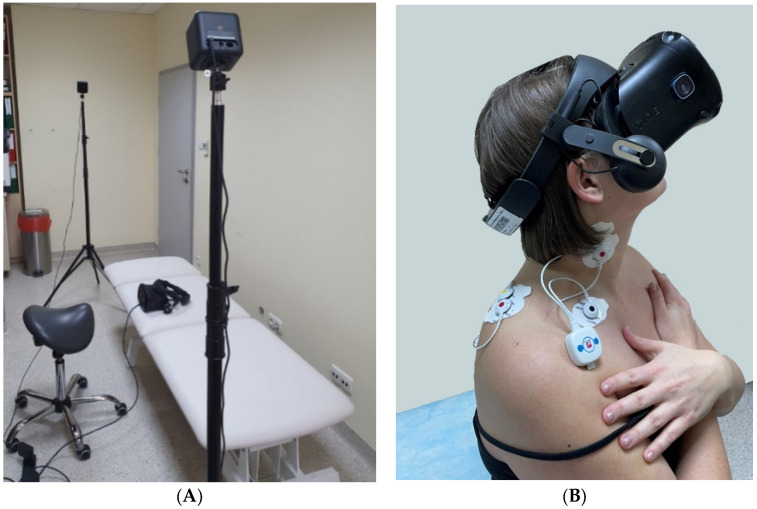
VRneck SOLUTION system: (**A**)—system setting; (**B**)—measurement of the muscle activity with VR goggles (HTC VIVE Cosmos Elite, HTC Corporation, New Taipei City, Taiwan) used to control functional movements.

**Figure 3:**
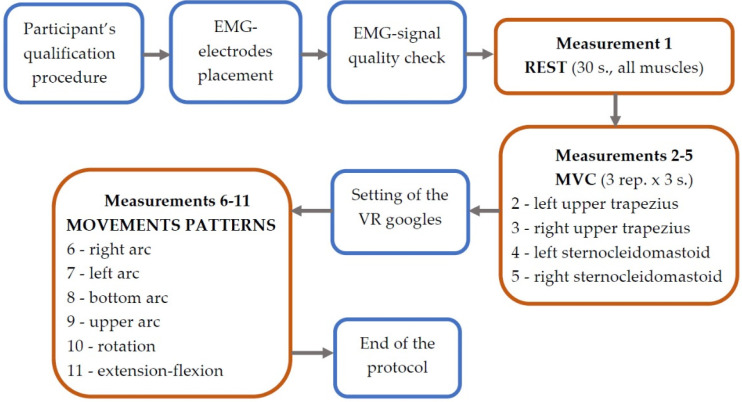
Study protocol.

In the beginning, the rest activity was tested during sitting in a comfortable position (measurement 1). Then, the maximum EMG activity of the three repetitions was recorded during the maximum voluntary contraction (MVC) procedures, sequentially for the left and right upper trapezius muscles and the left and right sternocleidomastoid muscles (measurements 2–5). MVC for the upper trapezius was recorded during shoulder elevation with extension, lateral flexion and contralateral rotation of the head. MVC for sternocleidomastoid was recorded during contralateral rotation with slight flexion of the head. All MVC measurements were collected during isometric contractions against the manual resistance of the therapist. Afterwards, six functional movements of the neck were tested, from which two were asymmetrical: right arc (measurement 6) and left arc (measurement 7); two were symmetrical in shape, but asymmetrical in movement direction: bottom arc (measurement 8) and upper arc (measurement 9); and two were symmetrical: rotation (measurement 10), and extension–flexion (measurement 11). Each trajectory (Figure 1) started from the centre (black dot), then the patient moved to the right/upwards, where they reached the maximum range of motion (green dot), and then to the opposite side as far as possible (red dot) and returned to the centre (black dot). Motion patterns (measurements 6–11), including path, range and speed, were controlled using a prototype augmented virtual reality system for neck diagnostics—the VRneck SOLUTION system (Consortium UMed and Edventure Research Lab. sp. z o.o., Lodz, Poland) (Figure 2), including VR googles (HTC VIVE Cosmos Elite, HTC Corporation, New Taipei City, Taiwan). The weight of the goggles was approx. 760 g. All the data were collected in a comfortable sitting position on a hooker with proper head posture controlled by a physiotherapist, and the order of the tested movements was the same for all patients. The rest activity and MVC measurements were collected without VR goggles. The protocol of data acquisition is presented in Figure 3.

### 2.3. EMG Data Analysis

**Figure 4:**
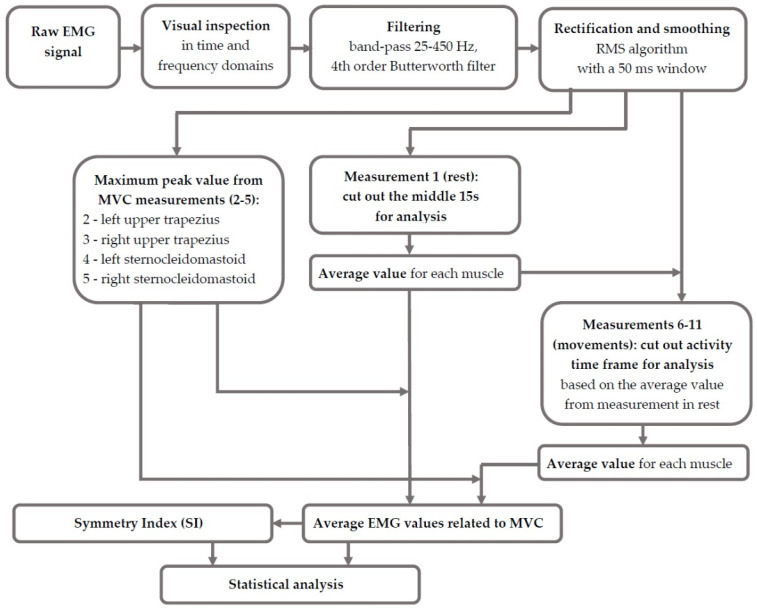
EMG data analysis protocol.

The Protocol of EMG data analysis is presented in Figure 4. Firstly, for each trial, unprocessed signals from SCM and UT muscles were visually inspected in time and frequency domains and detrended. EMG signals were then band-passed at 25–450 Hz, with a fourth-order Butterworth filter. Next, EMG signals were full-wave rectified and smoothed by using the RMS algorithm with a 50 ms window. Subsequently, EMG signals from all trials were normalized as the percentage of maximal voluntary contraction (% MVC). MVC was the maximum peak for the signal from measurement 2 (left upper trapezius), measurement 3 (right upper trapezius), measurement 4 (left SCM) and measurement 5 (right SCM) processed according to the above-described procedure.

Next, the movement was defined as a time frame when muscle activity from measurements 6–11 was higher than the average signal from baseline (measurement 1). Mean baseline signals (from measurement 1) and mean values of muscle activation during movement (data from movement time frame from measurements 6–11) related to MVC (from measurements 2–5) were taken for analysis. Moreover, for trials 1 and 6–11, for mean activation of SCM and UT muscles assessed for the right (*R*) and left (*L*) sides separately, symmetry indexes were calculated according to the following formula [27,28]:

$$SI=\frac{\left|X_{L}-X_{R}\right|}{0.5\cdot \left(X_{L}+X_{R}\right)}\cdot 100\%.$$

The *SI* factor is a method of percentage assessment of the differences between the parameters for both muscle activations. The *SI* = 0 indicates symmetry, while *SI* ≥ 100% indicates its asymmetry [27]. EMG signal processing was carried out in MATLAB software v. R2021a (MathWorks, Natick, MA, USA).

### 2.4. Statistical Analysis

Statistical analysis was performed using the PQStat 2021 software v. 1.8.2.238 (PQStat Software, Poznan, Poland). The normality of distribution was tested using the Shapiro–Wilk test and showed distributions different from normal in a few cases.

For the baseline signal, Student’s *t*-test for dependent samples and the Wilcoxon test in the absence of normal distributions were used. The level of significance was set at *p* ≤ 0.05. The same analysis was applied for mean muscle activation related to MVC within each motion separately. In this case, the same muscles were compared between the right and left sides.

Moreover, a non-parametric Friedman ANOVA with a post hoc test of Dunn Bonferroni and one-way ANOVA with post hoc Tukey HSD were used to find statistically significant differences between trials for specific muscles.

## 3. Results

### 3.1. Baseline Muscle Activity and Its Symmetry

**Figure 5:**
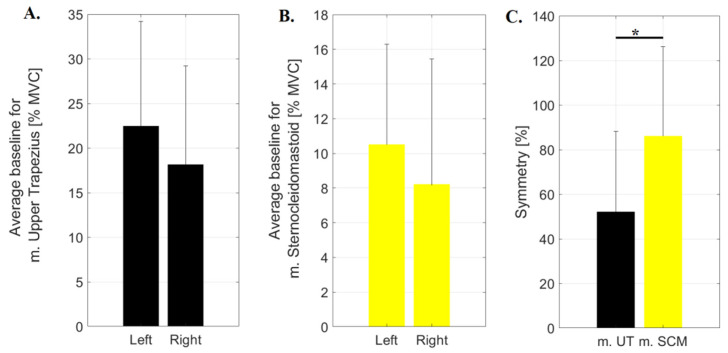
The values of the average activity recorded for the baseline normalized to the MVC for (**A**)—upper trapezius muscle; (**B**)—sternocleidomastoid muscle and (**C**)—values of symmetry index calculated for upper trapezius (m. UT) and sternocleidomastoid (m. SCM); * *p* ≤ 0.05.

Here, the different from normal distribution was only found for the average activity of the right sternocleidomastoid muscle. Following the application of Student’s *t*-test for dependent samples (UT) and the Wilcoxon test (SCM), no statistically significant differences were obtained between the mean baseline activities of these muscles normalized on MVC. However, it was noted that the activity of both muscles located on the left side of the neck was 23.74% and 27.88% higher for the upper trapezius and sternocleidomastoid, respectively (Figure 5A,B). A significantly higher (*p* = 0.0165) asymmetry was noted for SCM, where the SI index reached 86.14 ± 44.03% as compared to the values recorded for UT (52.18 ± 36.02%) (Figure 5C).

### 3.2. Muscle Activity during Motions

The results of the mean muscle activation related to MVC and the results of the *t*-test and Wilcoxon test are shown in Table 2.

#### 3.2.1. Right and Left Arc Motions

For motion along the right arc, distributions different from normal were found for the activity of the right SCM. For motion along the left arc, the different from normal distribution was only for the left SCM.

Following the Wilcoxon test, it was shown that for the right arc motion, the mean activity values of the left SCM muscle were significant, 127% higher than the average activity of the right SCM muscle. Following the *t*-test, it was shown that for the same motion, the mean activity values of the left UT muscle were significant, 53% higher than the average activity of the right UT muscle.

No statistically significant differences were shown for motion along the left arc. It is worth noting that the situation in this motion was the opposite of the previous one. Non-significantly higher values were for the average activity of the right UT and SCM muscles compared to the left UT and SCM.

#### 3.2.2. Bottom and Upper Arc Motions

For motions along bottom and upper arcs, the activities of the analysed muscles had normal distributions.

After applying the *t*-test for muscle activation along the bottom arc, it was shown that that the left SCM muscle has significantly (*p* = 0.0295) higher average activity than the right. The activity of the left SCM muscle was 87% higher than that noted for the right SCM muscle. No statistically significant differences were found for the UT muscle. Although, it is worth noting that it kept the trend of higher left activity by 32.5% with respect to that recorded for the right one.

For motions along the upper arc, no statistically significant differences were found between the mean activities of the evaluated muscles. In this case, no significantly higher activities were recorded for muscles lying on the right side of the neck.

#### 3.2.3. Rotation and Extension–Flexion Motions

For rotation and extension–flexion motions, different from normal distributions were recorded for the right SCM.

For the rotation motion, significantly higher activity values by 84.98% were recorded for the left SCM relative to the right one. The same but insignificant trend was maintained by the UT muscle, where the left side activity exceeded that of the right side by 14.45%.

For the extension–flexion motions, no statistically significant differences in muscle activity were noted.

### 3.3. Symmetry of Muscle Activity during Motions

In each of the motions studied, symmetry indexes for SCM and UT muscle activity had a distribution that was different from normal. Therefore, in each case, the Wilcoxon test was used to evaluate the differences between them.

It is worth noting that in each of the motions studied, the asymmetry value was significantly higher for the sternocleidomastoid muscle (Table 3). The highest differences between the symmetry indices were recorded for motion on the right arc, bottom arc, rotation, extension–flexion and motion on the left arc and the upper arc, sequentially.

### 3.4. Comparison of the Muscle Activity and SI between Motions

#### 3.4.1. Upper Trapezius Activity

Following a one-way ANOVA for the average activity of the left upper trapezius muscle, there were statistically significant differences between considered trials: F(10, 202) = 7.0764; *p* = 0.0001.

**Figure 6:**
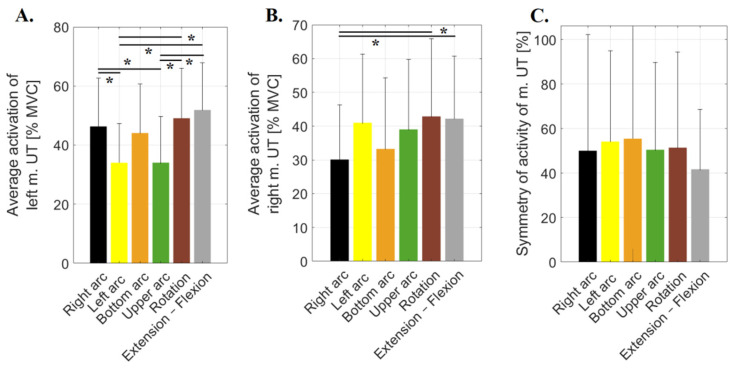
Average values of muscle activity recorded during six motions (right arc, left arc, bottom arc, upper arc, rotation and extension–flexion) for (**A**)—left trapezius muscle (m. UT), (**B**)—right trapezius muscle and (**C**)—symmetry index calculated for trapezius muscle during the analysed motions; * *p* ≤ 0.05.

After applying Tukey’s post hoc test, there were statistically significant differences between average muscle activity during the extension–flexion motion compared to mean activity during left arc motion (*p* = 0.0204) and upper arc motion (*p* = 0.0208) (Figure 4A). The left UT average activity was 52.44% and 52.31% higher during the extension–flexion motion, respectively. In addition, there were statistically significant differences between this muscle average activity during rotation compared to average activity during left arc motion (*p* = 0.0043) and upper arc motion (*p* = 0.0044) (Figure 6A). The left UT activity was 102% and 32% higher in the rotation motion, respectively. Moreover, significantly higher activity of this muscle during the right arc was noted compared to the activity during left arc motion (*p* = 0.0358). However, its activity during the right arc motion was significantly lower (*p* = 0.0365) than that noted during the upper arc motion. The activity of the left UT was the highest for the extension–flexion (51.87 ± 20.12%) and rotation motions (49.08 ± 16.96%) and the lowest during the left (34.03 ± 13.25%) and upper arc motions (34.06 ± 15.60%).

In the case of the right upper trapezius muscle, there were statistically significant differences between the two trials after performing a one-way ANOVA (F(10, 202) = 3.2017; *p* = 0.0107). Its significantly lower activity was recorded during right arc motion versus that noted during rotation (*p* = 0.0309) and extension–flexion motion (*p* = 0.0475) (Figure 6B). The activity of the right UT was the highest for the rotation motion (42.88 ± 25.88%) and extension–flexion motion (42.21 ± 18.52%) and the lowest along right arc motion (30.10 ± 16.26%).

The smallest asymmetry was observed for the extension–flexion motion and the highest for the lower arc motion. However, there were no statistically significant differences between the symmetry indices calculated for the UT for the analysed trials (Figure 6C).

#### 3.4.2. Sternocleidomastoid Muscle

**Figure 7:**
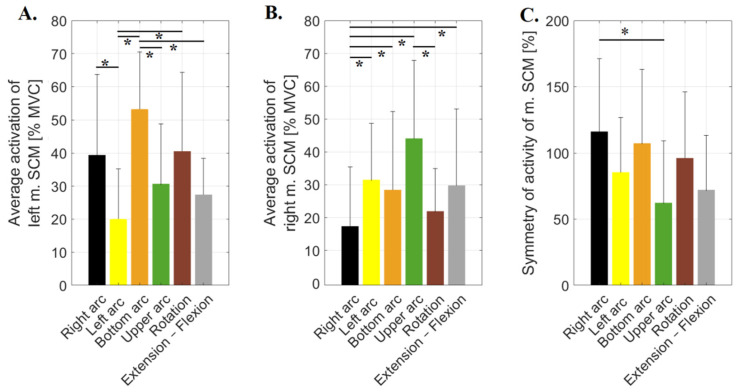
Average values of muscle activity recorded during six motions (right arc, left arc, bottom arc, upper arc, rotation and extension–Flexion) for (**A**)—left sternocleidomastoid muscle (m. SCM), (**B**)—right sternocleidomastoid muscle and (**C**)—symmetry index calculated for the sternocleidomastoid muscles during the analysed motions; * *p* ≤ 0.05.

Following the Friedman ANOVA, it was shown that, for the left sternocleidomastoid activity, there are statistically significant differences between the trials: F(5, 108) = 41.8210; *p* = 0.0001. The highest activity of this muscle was recorded for motion along the lower arc. After applying the Dunn–Bonferroni post hoc test, it was shown that the activity of this muscle in this motion was significantly higher than during extension–flexion (*p* = 0.0076), upper arc motion (*p* = 0.0038) and along the left arc motion (*p* = 0.0001). In addition, the average activity of this muscle along the left arc was the lowest. It was significantly lower than that recorded along the right arc motion (*p* = 0.0001) and rotation (*p* = 0.0009) (Figure 7A).

Following the Friedman ANOVA, it was shown that for the right sternocleidomastoid activity, there are statistically significant differences between the trials: F(5, 108) = 32.7476; *p* = 0.0001. The lowest activity of this muscle was recorded for the motion along the right arc. After applying the Dunn–Bonferroni post hoc test, it was shown that the activity of this muscle in this motion was significantly lower than during extension–flexion (*p* = 0.0106), and motion along the upper arc (*p* = 0.0001), lower arc (*p* = 0.0146) and the left arc (*p* = 0.0006). In addition, it was shown that the average activity of this muscle in motion along the upper arc was the highest. It was significantly higher than that recorded for rotation (*p* = 0.0273) (Figure 7B).

Following the Friedman ANOVA, it was shown that for the SI index calculated for sternocleidomastoid activity, there are statistically significant differences between the trials: F(5, 108) = 17.2204, *p* = 0.0041. After applying Dunn–Bonferroni’s post hoc test, statistically significant differences were shown between SIs calculated for the right and upper arcs (*p* = 0.0273) (Figure 7C). In these cases, the SI was 86% higher during right arc motion, showing the highest asymmetry in this motion. The lowest asymmetry was noted for upper arc motion (62.36 ± 46.92).

## 4. Discussion

In this paper, the activity of the upper trapezius and sternocleidomastoid muscles during six functional neck motions was analysed, as well as the symmetry of muscle activation. It was shown that the resting activity of both muscles located on the left side of the neck was 23.74% and 27.88% higher than for the right side for the upper trapezius and sternocleidomastoid muscles, respectively. Significantly higher asymmetry was noted for the SCM, where the SI index reached (86.14 ± 44.03)% compared to the values recorded for UT (52.18 ± 36.02)%. The highest asymmetries during motion were for the sternocleidomastoid for motion along the right arc (116.19 ± 55.07)% and the lowest for motion along the upper arc (62.36 ± 46.92)%. For the upper trapezius, the asymmetries were smaller. The highest was for motion along the lower arc (55.47 ± 50.87)% and the lowest was for flexion and extension (41.75 ± 27.00)%. A high degree of asymmetry is evident when analysing the behaviour of the left and right UT muscles in six functional motions because all of them were at a similar level. Nevertheless, there are a lot of significant differences in the activity of the left UT muscle within the functional motions. However, the UT muscle located on the right side of the neck was activated similarly for all movements. It is noteworthy that for both sides, the motions of extension–flexion and rotation caused the highest activity of this muscle in relation to the activity observed in the other movements. For the SCM muscle, the level of asymmetry between movements was much more marked than for the UT muscle. The movements causing its significant asymmetry were those along the right and lower arcs. The lowest asymmetry was recorded for movement along the upper arc.

It is worthful to notice that the activity of both muscles at rest was relatively high. Villanueva et al. [29] reported values of 2.2–3.1% of MVC in UT muscle while sitting in front of a computer screen in healthy participants, which is much less than the results of our study. Presently (25 years later), significantly more people are sitting in front of a computer and using other electronic devices such as smartphones and tablets, mostly from a young age, which causes forward head posture [30]. The anterior position of the head to the gravity line causes muscular imbalances affecting both deep (e.g., suboccipital, scalenus anterior and semispinalis) and superficial neck muscles (e.g., upper trapezius, sternocleidomastoid and levator scapulae) [30]. Increased activity of UT muscles at rest can compensate for weakened cervical extensor muscles [30], and this was confirmed by other research [16,31,32,33], as well as in our study. Conversely, according to Lee, Han, Cheon, Park and Yong [22] the activity of the neck muscles, including the upper trapezius and the sternocleidomastoid, is decreased. Such mechanisms, showing disturbed activation of the neck muscles, are observed even in the “healthy” population, being one of the reasons for the rapidly growing rate of neck disorders [34]. The presence of increased or decreased muscle activity, as well as its asymmetry, was confirmed in our study. Additionally, mental or physical stress can increase muscle activity, which was proven by Luedtke et al. [35] in the upper trapezius. The rest activity of the measured muscles in the study of Khan, Khan, Bhati and Hussain [30] was much lower than in our study (1.4–2.6% MVC), while the activity during motions was over 50% MVC in the group with proper head posture and over 70% in participants with forward head posture and was higher than the values achieved in our study. These differences could be caused by the different methodologies of the sEMG measurement, especially different movements performed to assess muscles during motion. Additionally, lower activation during the MVC procedure, caused by muscle shortness [33], position [32] or other factors, could result in relatively high values of muscle activation at rest in our study, as it was given as a percentage. Decreased neuromuscular efficiency in the superficial muscles of the neck, such as the sternocleidomastoid during isometric neck flexion, was proved by Falla, Bilenkij and Jull [13]. Another reason for the high rest activity of measured muscles could be incomplete or incorrect relaxation during this measurement.

Higher activity of the left upper trapezius and sternocleidomastoid muscles at rest, as well as in some symmetrical movements such as extension–flexion movement (SCM and UT) and rotation (UT), can be caused by lateralization of the body. All the participants included in this study were right-handed. The difference in the UT and SCM muscle activity was also observed by Wang and Liu [36] during bilateral hanger reflex, as well as without it. In their study, muscles on the right side of the body were more active, although they did not provide information about the lateralization of the participants and did not analyse these differences. In contrast, Khan, Khan, Bhati and Hussain [30] showed similar activity of UT and SCM muscles on both sides during rest and activity in the population with and without forward head posture. In their study, participants’ lateralization was also not described. So far, there have been no studies concerning the influence of body lateralization on neck muscle activity. Another factor that could influence the higher activity of the left upper trapezius and left sternocleidomastoid during rotational movements was the direction of the movement, as it started from the right rotation, and once activated, the muscle remained active throughout the whole movement. Błaszczyk and Ogurkowska [37] confirmed the existence of contralateral muscle imbalances in the lumbar spine during transferring of the load. So far, no other publications concerning the symmetry of neck muscle activation have been found.

Analysis of the activation of both assessed muscles during movements showed high activity during rotation and extension–flexion movements. Both these muscles are primarily responsible for neck rotation when working unilaterally and for neck flexion (sternocleidomastoid) or extension (upper trapezius) when working bilaterally [9,10]. Additionally, as superficial muscles working on a larger moment arm than deep muscles, they are responsible for starting the movement and its range [12]. These facts could be the reason why the activity of these muscles is higher in simple movements such as extension–flexion and rotation. Against, deep neck muscles could be probably more active during arc motions, which require more precision to follow the pattern. When concerning the activation of the UT and SCM muscles and their symmetry across different movements, it seems that the extension–flexion movement was the most symmetrical one for both measured muscles. Additionally, low SI values were noticed for the upper arc movement for the SCM muscle, although it does not activate the left and right muscles simultaneously. These results show that these two movements can be the best to assess the symmetry of neck muscles’ activation. In our opinion, asymmetrical movements such as right/left arcs give results that are difficult to interpret due to the fact that they can be performed with different speeds, giving different muscle activity results.

Practical applications of this study include the possibility to use the above-described results to define reference values for patients with neck pain. These data can be used to evaluate the effectiveness of the therapy, as is planned in the VRneck SOLUTION project, as well as to set the goal of neck therapy. Our results may be the cause of discussion about the physiological asymmetry of spinal muscle activation and the influence of body lateralization on the symmetry of muscles’ activation, as well as the physiological effects of changes in body posture such as forward head posture, which have been observed in the last 20 years.

Some limitations of this study have to be acknowledged. Firstly, all the participants performed all movements in the same direction (firstly to the right or upwards, and then to the left or downwards and back to the starting position), which could have influenced the muscle activity. Additionally, the fact that all the participants were right-handed could have influenced the results. Secondly, the BMI values showed that they had normal weight or were slightly overweight. Bartuzi et al. [38] showed that the sEMG signal is sensitive to the fat tissue layer. However, subcutaneous tissue in the neck region contains little fat and therefore should not significantly influence the EMG signal. Thirdly, high rest activity, shown in this study, needs to be verified in other positions, i.e., in lying. The habitual postures of the participants were also not assessed, although the measurement was performed in the proper head posture. Fourthly, the weight of the VR goggles could influence the results of the activity assessment during motions, especially by increasing activation of the upper trapezius muscles. Nevertheless, high activity of these muscles was also confirmed in rest conditions before the application of VR goggles. In future studies, it should be worthwhile to perform the movements in non-immersive virtual reality to avoid the usage of additional head-mounted equipment. Lastly, this study comprised analysis of sEMG signals from only 18 people with a wide age spread (24–68 years of age). Diversity in muscle activation may be relatively high in a healthy population. In further studies, more people should be examined to define “normal values” of symmetry in neck muscles’ activity. It would be also worthwhile to compare above-described results to the group with neck pain to understand the mechanisms of changes in muscle activation during rest and movement. Further analyses are also necessary to determine muscle activation patterns.

## 5. Conclusions

This study compared the activation of the upper trapezius and sternocleidomastoid muscles at rest and during basic motions of the neck to assess the symmetry of muscle activation. Extension–flexion movement was the most symmetrical one due to the fact that it involves simultaneous, bilateral activation of measured muscles. Therefore, this movement can be useful to assess the symmetry of neck muscles’ activation. The results presented in this manuscript showed that there is higher activity of the muscles on the left side, which can be connected to the lateralization of the body. Additionally, high activity of the muscles at rest suggests improper upper body posture and/or incomplete relaxation during the measurement. Further studies are required to verify the above-presented results, determine muscle activation patterns and compare healthy people to patients with neck pain.

## Figures and Tables

**Table 1:** Characteristics of the participants.

Group	Age (Years) (Mean ± SD)	Body Mass (kg) (Mean ± SD)	Body Height (cm) (Mean ± SD)	Body Mass Index (kg/m^2^) (mean ± SD)	Neck Disability Index (Points) (Median, Quartiles Q1 and Q3)
N = 18 (9 males, 9 females)	42.5 ± 13.3	76.9 ± 15.9	173.5 ± 10.5	25.4 ± 3.8	2.0 Q1 = 1, Q3 = 4

**Table 2:** Statistically significant differences for *t*-test (^T^) and Wilcoxon’s (^W^) test for dependent groups in mean muscle activity, *p* < 0.05.

Motion	Left UT [% MVC]	Right UT [% MVC]	Left SCM [% MVC]	Right SCM [% MVC]	*p*-Value
Right arc	46.30 ± 16.35	30.10 ± 16.26	39.39 ± 24.35	17.35 ± 18.03	Left UT-Right UT, *p* = 0.0035 ^T^ Left SCM-Right SCM, *p* = 0.0249 ^W^
Left arc	34.03 ± 13.25	40.99 ± 20.35	19.98 ± 15.14	31.50 ± 17.21	-
Bottom arc	44.07 ± 16.55	33.26 ± 21.03	53.21 ± 25.01	28.43 ± 23.89	Left SCM-Right SCM, *p* = 0.0295 ^T^
Upper arc	34.06 ± 15.60	39.02 ± 20.64	30.69 ± 18.08	44.10 ± 23.70	-
Rotation	49.08 ± 16.96	42.88 ± 25.88	40.53 ± 23.83	21.91 ± 13.01	Left SCM-Right SCM, *p* = 0.0311 ^W^
Extension- flexion	51.87 ± 20.12	42.21 ± 18.51	27.43 ± 10.98	29.78 ± 23.34	-

**Table 3:** Mean and standard deviation of symmetry indices calculated for upper trapezius (UT) and sternocleidomastoid (SCM) muscles, where ↑ denotes the percentage of positive difference in the symmetry index for SCM compared to that recorded for UT; * denotes statistically significant differences, *p* < 0.05.

Motion	SI–UT	SI–SCM	↑; *p*-Value
Right arc	50.07 ± 52.20	116.19 ± 55.07	132%; 0.0006 *
Left arc	54.19 ± 40.90	85.46 ± 41.38	57.7%; 0.0480 *
Bottom arc	55.47 ± 50.87	107.37 ± 55.65	93.54%; 0.0152 *
Upper arc	50.53 ± 39.27	62.36 ± 46.92	23.40%; 0.4112
Rotation	51.46 ± 43.05	96.17 ± 50.04	86.88%; 0.0132 *
Extension-flexion	41.75 ± 27.00	72.14 ± 41.18	72.76%; 0.0306 *

## Data Availability

The measurement data used to support the findings of this study are available from the corresponding author upon request.
